# Non-invasive multispectral optoacoustic tomography resolves intrahepatic lipids in patients with hepatic steatosis

**DOI:** 10.1016/j.pacs.2023.100454

**Published:** 2023-01-20

**Authors:** Nikolina-Alexia Fasoula, Angelos Karlas, Olga Prokopchuk, Nikoletta Katsouli, Michail Bariotakis, Evangelos Liapis, Anna Goetz, Michael Kallmayer, Josefine Reber, Alexander Novotny, Helmut Friess, Marc Ringelhan, Roland Schmid, Hans-Henning Eckstein, Susanna Hofmann, Vasilis Ntziachristos

**Affiliations:** a Chair of Biological Imaging at the Central Institute for Translational Cancer Research (TranslaTUM), School of Medicine, Technical University of Munich, Germany; b Institute of Biological and Medical Imaging, Helmholtz Zentrum München, Neuherberg, Germany; c Department for Vascular and Endovascular Surgery, Klinikum rechts der Isar, Technical University of Munich, Munich, Germany; d DZHK (German Centre for Cardiovascular Research), partner Site Munich Heart Alliance, Munich, Germany; e Department of Visceral Surgery, Klinikum rechts der Isar, Munich, Germany; f Institute for Diabetes and Regeneration Research, Helmholtz Zentrum München, Neuherberg, Germany; g Department of Internal Medicine II, Klinikum rechts der Isar, Technical University of Munich, Munich, Germany; h Department of Internal Medicine IV, Klinikum der Ludwig Maximilian University of Munich, Munich, Germany

**Keywords:** Photoacoustics, MSOT, Lipid imaging, Non-alcoholic fatty liver disease, NAFLD, Molecular imaging, Metabolism

## Abstract

Hepatic steatosis is characterized by intrahepatic lipid accumulation and may lead to irreversible liver damage if untreated. Here, we investigate whether multispectral optoacoustic tomography (MSOT) can offer label-free detection of liver lipid content to enable non-invasive characterization of hepatic steatosis by analyzing the spectral region around 930 nm, where lipids characteristically absorb. In a pilot study, we apply MSOT to measure liver and surrounding tissues in five patients with liver steatosis and five healthy volunteers, revealing significantly higher absorptions at 930 nm in the patients, while no significant difference was observed in the subcutaneous adipose tissue of the two groups. We further corroborated the human observations with corresponding MSOT measurements in high fat diet (HFD) - and regular chow diet (CD)-fed mice. This study introduces MSOT as a potential non-invasive and portable technique for detecting/monitoring hepatic steatosis in clinical settings, providing justification for larger studies.

## 1 Introduction

Hepatic steatosis is a benign condition associated with chronic inflammation and may lead to non-alcoholic steatohepatitis (NASH), fibrosis [1], [2], [3], cirrhosis, or liver cancer [4], [5]. There is therefore a need to swiftly assess steatosis to prompt early interventions, such as dietary and lifestyle changes or drug treatments, as well as to assess the efficacy of these interventions [6].

Today, monitoring of hepatic steatosis is primarily based on liver biopsies [7], which are analyzed for lipid content [3], [8]. However, biopsies are limited by their invasive nature, pain, and overall patient inconvenience and are not appropriate for disseminated observations or longitudinal monitoring. Moreover, biopsies are prone to sampling errors, may lead to complications, such as hemorrhages [8], and come with considerable cost when considering the time and infrastructure required for a diagnostic test. With steatosis becoming more prevalent worldwide as obesity rates increase, there is a clear need for non-invasive lipid assessment in liver [6].

Imaging techniques have been considered for the non-invasive assessment of liver lipid content [9], [10]. X-ray computed tomography (CT) can resolve and quantify intrahepatic lipids and allow for staging of hepatic steatosis but is characterized by low sensitivity in mild cases [11] and uses ionizing radiation that limits frequent monitoring. Magnetic resonance (MR) imaging and spectroscopy techniques offer higher sensitivity and specificity in assessing hepatic steatosis, without using ionizing radiation [11], [12], [13]. Conversely, MR techniques require expensive and low-throughput infrastructure that is not appropriate for disseminated use.

Ultrasound (US) based techniques have been considered as a portable and low-cost alternative for assessing lipid content. While conventional ultrasonography is not sensitive to lipids [9], indirect measurements of mechanical properties using ultrasound elastography (USE) have shown potential to provide a surrogate marker for steatosis, as they associate with stiffness changes due to lipid accumulation [9], [10], [13], [14], [15]. In particular, controlled attenuation parameter ultrasound (CAP-US) has been studied for diagnosing hepatic steatosis, achieving detection sensitivity in the 80% range, but possibly with performance that is operator - dependent [16], [17]. Nevertheless, stiffness is not a parameter that is lipid specific and may be affected also by other pathologies, limiting the wide application of CAP-US in the general population.

Optoacoustic imaging resolves optical contrast in tissue with resolutions that are similar to ultrasonography. Multispectral optoacoustic tomography (MSOT) is a portable and non-invasive technique that has been employed to visualize hemodynamics, tissue oxygenation, and inflammation in humans and animals using illumination wavelengths between 700 and 900 nm [18], [19], [20], [21], [22], [23], [24], [25], [26]. Nevertheless, recent observations indicate that in vivo visualization of lipids is possible with MSOT by illumination on or around 930 nm [21], [23], [26], [27], [28], where lipids exhibit a characteristic strong absorption peak. Since previous publication has already presented a histological validation of MSOT imaging of hepatic steatosis in mice [29], extending lipid-detection with MSOT to the human liver could significantly impact the monitoring and management of hepatic steatosis in humans by potentially allowing frequent, non-invasive assessment of the liver’s lipid content. However, reliable detection of lipids in the liver would require penetration depths of a few centimeters, despite the organ being surrounded by deformable tissues.

Here, we present a pilot study to examine whether MSOT can detect lipids at such depths within the human liver by their strong absorption around 930 nm. To this end, we employed a hybrid MSOT–US modality and developed an imaging protocol and data analysis methodology that leverages the availability of ultrasound images co-registered with optoacoustic images to offer image-guided recording of multispectral data from the liver. This feature allows for accurate comparisons of the absorption spectra in the near infrared (NIR, 700–970 nm) between subjects, from the liver and the overlying subcutaneous fat (SAT). A particular feature of the data analysis protocol used herein is the use of the SAT spectrum as a reference spectrum to account for system variations and depth-dependent attenuation effects on the spectra collected from the liver, thereby increasing the accuracy of the observation.

To further investigate the accuracy and validity of the approach, we conducted a control study in animals, using mice with induced steatosis and healthy mice. The goal of the animal study was to validate the spectral features obtained from different depths in humans using signals from more shallow depths, i.e., using measurements that are not affected by the signal dependence of the optoacoustic measurement to depth, due to light attenuation. In both human and animal studies we observed strong optoacoustic signals at 930 nm for the lipid-rich tissue compartments, such as SAT, confirming that the 930 nm peak can be used for examining lipid content in human liver and that depth dependent attenuation did not significantly affect the spectral appearance of the data collected. In contrast, the 930 nm peak was not prominent in the livers of healthy mice and healthy volunteers. Therefore, the investigation gives preliminary support to MSOT detecting elevated lipid levels in the livers of patients with hepatic steatosis, setting the stage for larger clinical studies.

## 2 Methods

### 2.1 Human imaging

Five patients with previously diagnosed liver steatosis (n_1_ = 5, 3 males and 2 females, mean age 58, range 46–61, mean BMI= 27.5 kg/m^2^, range 21.5–32) were included in this pilot study. Liver steatosis in the patients was diagnosed using ultrasound imaging, CT, and visual inspection of the liver during an open abdominal surgery. Two out of the five patients had grade 2 liver steatosis while the other three patients had grade 3.

Two out of five patients were overweight (BMI > 25 kg/m^2^) and two of them had class I obesity (BMI > 30 kg/m^2^). Three patients had a history of hyperlipidemia, two patients had a history of hypertension, and one patient had type 2 diabetes.

Both diagnosis and grading of liver steatosis was performed using ultrasound features that include liver brightness, contrast differences between the liver and the kidney, ultrasound appearance of the intrahepatic vessels, liver parenchyma and diaphragm [30], [31]. Since ultrasonography provides nowadays accurate detection of moderate to severe hepatic steatosis [30], an additional biopsy was not necessary. Moreover, in one out of five patients, hepatic steatosis was primarily diagnosed during open abdominal surgery (esophagectomy for adenocarcinoma of the esophagogastric junction) and described macroscopically as severe liver steatosis. In the other four patients included, hepatic steatosis was primarily diagnosed by a primary care physician and validated after inclusion in this study. In two patients, liver steatosis was additionally described in CT.

Additionally, five healthy volunteers (n_2_ = 5, 3 males and 2 females, mean age 31, range 24–38 mean BMI= 20.5 kg/m^2^, range 19.7–21.6), were recruited as control subjects. All volunteers were non-smokers and had no history of cardiovascular or metabolic disease. Participants signed an informed consent form prior to enrollment in the study, which was approved by the ethics committee of the medical faculty of the Technical University of Munich (Protocol No 520/19 S).

**Fig. 1:**
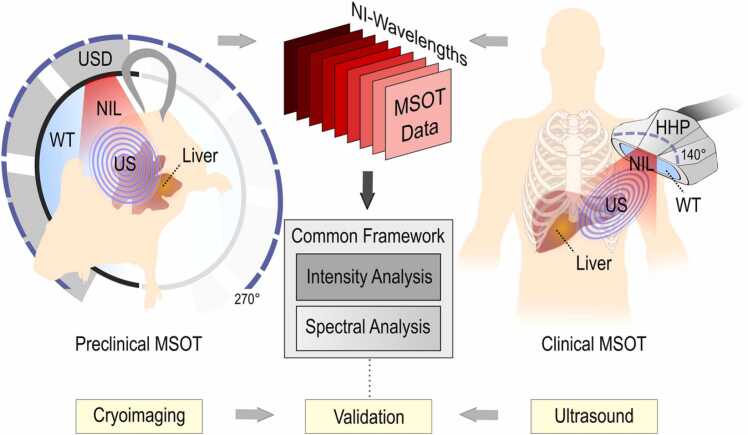
MSOT principle of operation and common analysis framework. Left: Preclinical MSOT imaging of the liver and validation by cryoimaging. Right: Clinical MSOT imaging of the liver and validation by ultrasound. The common analysis framework includes the intensity and spectral analysis. USD: Ultrasound detector, NIL: Near-infrared light, US: Ultrasound, WT: Water tank, HHP: Hand-held probe, NI: Near-infrared. The coverage angle in preclinical MSOT is 270° and in hand-held clinical MSOT 140°.

Optoacoustic scans took place in a quiet room with a normal room temperature (≈23 °C). Subjects were asked not to consume any food, alcohol, or caffeine for 8 h before the MSOT examination. Both participants and examiners wore laser safety goggles. All participants were asked to lie in a supine position. For both patients and healthy volunteers, the hand-held scanning probe was placed over the right hypochondriac region in a transversal position identified by anatomic knowledge under real-time ultrasound imaging (Fig. 1). Each scan lasted for approximately 60 s. The total duration of the MSOT examinations was approximately 5 min (patient and system preparation included), comparable to standard ultrasound examinations. This hand-held MSOT is capable of imaging up to depths of approximately 3–4 cm, which is sufficient to reach the liver.

### 2.2 Animal imaging

The Government of Upper Bavaria approved all procedures involving animal experiments. To induce substantial obesity and steatosis, mice (n_3_ = 5 B6 (Cg)-Tyr^c-2 J^/J, 3 males and 2 females; Charles River, River Laboratories, Wilmington, MA, USA) were fed with a high‐fat, high‐sugar diet (HFD) comprising 58% kcal from fat (D12331; Research Diets, New Brunswick, NJ, USA) for 20 weeks. Another age-matched cohort of mice (n_4_ = 5 B6 (Cg)-Tyr^c-2 J^/J, 2 males and 3 females) were fed a regular chow diet (CD) as a control. The mice were housed on a 12:12–h light–dark cycle at 22 °C with free access to water and food.

All animals were anesthetized by inhalation of 2% isoflurane (Zoetis GmbH, Berlin, Germany) delivered in combination with oxygen. Animals were positioned in the MSOT machine according to a standard measurement protocol [20]; after being placed into a thin and transparent polyethylene membrane, the mice were submerged in a water bath of 34 °C. The water ensures acoustic coupling and a stable body temperature. In vivo scans were conducted using an MSOT device tailored for small animal imaging described below [20]. Each mouse MSOT scan lasted for approximately 11 min recording data at many different positions along the long axis of the animal over the thorax and abdomen regions.

MSOT recordings were anatomically validated via cryoimaging, which was carried out after euthanizing the animals and freezing them to − 50 °C. The mice were next embedded in optimal cutting temperature compound (O.C.T, Tissue Teck, Sakura Finetek, USA) and transversely cryosliced at steps of 50 µm along their long axis using a customized cryoimaging system. The latter consisted of a commercial cryotome (CM 1950, Leica microsystems, Germany) equipped with a CCD camera (AndorLucaR© CCD camera (DL-604 M, Andor Technology, Belfast, UK), which recorded one RGB color image of the mouse cross-section after each slicing step. Acquired images were further processed (denoising, contrast enhancement, and geometric transformation) for visualization purposes.

### 2.3 Hand-held MSOT/US and mouse MSOT systems

MSOT scans of patients were conducted using a clinical hand-held MSOT-Ultrasound system (MSOT Acuity Echo©, iThera Medical GmbH, Munich, Germany) equipped with a handheld scanning probe. Briefly, the custom-built hand-held MSOT system is capable of acquiring real time optoacoustic images at a frame rate of 25 Hz and co-registered US images at a frame rate of approximately 9 Hz. The hand-held probe is enclosed within a light-weight 3D-printed casing and is equipped with 256 piezoelectric elements with a central frequency of 4 MHz arranged in an arc of 145^o^ for ultrasound detection. The cavity between the half-arc detector and specimen was filled with heavy water (D_2_O), which absorbs less light in the near-infrared range compared to normal water, while providing ideal coupling with the ultrasound transducer. Illumination was delivered in the form of short light pulses (approximately 10 ns in duration), at a rate of 25 Hz. For each pulse, almost 15 mJ of energy were delivered over a rectangular area of around 1 × 4 cm, which is well below the safety limits of laser use for medical applications [32]. For multispectral imaging, we acquired images at 28 different wavelengths (from 700 to 970 nm, 10 nm steps). While the 900–970 nm region was used to detect lipids, the spectral information in the 700–900 nm range was useful for identifying strongly absorbing structures such as large vessels that might obscure tissues of interest.

Live mice were scanned using a small animal MSOT device (inVision© 256-TF, iThera Medical GmbH, Munich, Germany). Technical details of the device have been described elsewhere [20]. In brief, mice were illuminated with near-infrared light pulses of 29 different wavelengths (680–960 nm at steps of 10 nm) at a repetition rate of 10 Hz and with pulse energy of approximately 80 mJ. A fiber bundle splitting configuration with multiple outputs ensured homogenous ring-shaped line illumination around the mouse body (Fig. 1). The ultrasound waves produced upon pulsed illumination were recorded by means of a 256-element-array of piezoelectric ultrasound detectors, covering an angle of 270° around the animal. A moving stage mechanism enabled the acquisition of full-thickness tomographic images of the mouse body at several positions along its long axis. Each scan included the recording (and averaging) of 10 frames at each of 23 different positions along the animal.

The employed human and mouse MSOT systems are based on the same principle of operation (single-pulse-per-frame, SPPF) providing tomographic images of the examined anatomic regions. As described above, the delivery of illumination and ultrasound detector arrays differ between the two setups, providing high adaptability and flexibility to the user depending upon application. In specific, the mouse system employs mechanical stages for scanning different parts of the animal while the hand-held configuration of the human system enables the examination of different body regions.

All acquired optoacoustic datasets were reconstructed using a model-based reconstruction algorithm developed in previous work [33] with non-negativity constraint, as usual in such studies.

### 2.4 Optoacoustic data analysis

The two systems employed herein share the engine and, after image acquisition, a common methodology was applied for data analysis in both humans and mice. Our analysis included the 29 wavelengths from 680 to 960 nm at steps of 10 nm for the mouse dataset and the 28 wavelengths from 700 to 970 nm at steps of 10 nm for the clinical dataset. A single characteristic set of 29 or 28 single-wavelength images, known as a ‘multispectral stack’, was selected for each mouse or human subject for further analysis. The selection criteria were an absence of motion and high image quality. Three different regions of interest (ROIs) based on the anatomical location in the co-registered ultrasound images were manually delineated for each multispectral stack: i) the liver region, ii) the subcutaneous fat (SAT) region, and iii) the entire imaged tissue region. The latter ROI included both the liver and SAT regions and was taken as the ‘background region’ (BGR) for each recorded image. The ROIs in the mouse data were manually segmented in consultation with two biologists with extensive experience in MSOT mouse imaging. The ROIs in the human data were manually segmented using both ultrasound data and in consultation with two clinicians with experience in clinical MSOT imaging.

As a next step, we adjusted the measured optoacoustic signals within the liver to account for differences arising from either i) exogenous factors, such as normal fluctuations of the laser energy per pulse or ii) subject-dependent factors, such as variations in the thickness, type, and perfusion of tissue layers in the scanned region. This adjustment was accomplished by first calculating the mean pixel intensity within the liver ROI and the BGR for the selected 29 (mouse) or 28 (human) single-wavelength images of all subjects. Then, the ROI/BGR ratio of the calculated mean values was extracted for each single-wavelength image. The extracted ratios were normalized against the maximum ratio value of each selected multispectral stack in order to fall within the range of [0,1]. By plotting the normalized ROI/BGR ratios for the measured wavelengths, we calculated for each subject a ‘normalized ratio spectrum’ in the NIR, which provided a common reference for all subjects and yielded comparable results. Next, the per-subject normalized ratio spectra were used to estimate the ‘mean’ normalized ratio spectra within the SAT and liver ROIs for the healthy and steatosis group in both mice and humans.

Furthermore, we compared the mean of the ROI/BGR ratio at 930 nm between the two groups (healthy and steatosis) in mice and humans to investigate possible correlations between the preclinical and clinical MSOT data of hepatic steatosis. This wavelength was chosen because it is where lipid absorption is highest in the NIR. The mean of the ROI/BGR ratio at 930 nm in healthy subjects and the same mean value in subjects with hepatic steatosis were compared using unpaired t-tests, since samples conformed to a normal distribution, as indicated by Shapiro-Wilk tests.

To further evaluate and quantify the capability of MSOT to detect liver steatosis at high, usual for human imaging, depths of several centimeters (> 2–3 cm) we performed a SNR analysis with increasing depth in the human MSOT data. To this end, the manually segmented liver regions in the 930 nm-MSOT images were further divided in 5 mm-thick horizontal segments and the SNR was calculated within the SAT segment and the resulted 5 mm-thick liver segments for all subjects (patients and healthy volunteers). The SNR for each segment at the various depths was calculated as the mean optoacoustic signal of the segment divided by the standard deviation of the noise, or else the signal measured within the region above the skin line in each image which corresponds to the water tank of the hand-held MSOT probe.

Data processing and statistical analysis, including the creation of figures, was performed with MATLAB (R2019a, MathWorks, 2019, Inc., Massachusetts, United States) and R (version 3.6.0, R Core Team, 2019, Vienna, Austria). The results are reported as the mean ± standard error of the mean. We also report p-values and effect sizes of the relative statistical tests.

## 3 Results

**Fig. 2:**
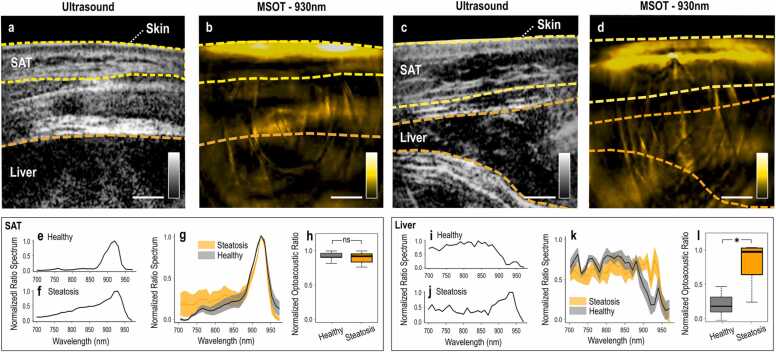
Human hand-held MSOT imaging and data analysis. (a) US image over the hypochondriac region of a healthy volunteer. (b) MSOT image recorded at 930 nm of the hypochondriac region of the same healthy volunteer. (c) US image of the liver of a patient with liver steatosis. (d) 930 nm-MSOT image of the same region of the patient. In (a)-(d): The dashed yellow lines delineate the skin/SAT layers and the dashed orange lines the liver region. (e-f) Normalized ROI/BGR ratio spectrum within the SAT region for a healthy volunteer and a patient with steatosis. (g) Mean (± standard error of the mean) normalized ROI/BGR ratio spectra for the whole cohort of healthy subjects (gray) and patients (orange). (h) Normalized ROI/BGR mean pixel intensity ratio at 930 nm within the SAT region for healthy volunteers (gray) and patients (orange). (i-j) Normalized ROI/BGR ratio spectrum within the liver region of a healthy volunteer (gray) and a patient (orange). (k) Mean (± standard error of the mean) normalized ROI/BGR ratio spectra for the whole cohort of healthy volunteers (gray) and patients (orange). (l) Normalized ROI/BGR mean pixel intensity ratio at 930 nm within the liver region of healthy subjects (gray) and patients with liver steatosis (orange). ns = non statistically significant, *p < 0.05.

We first conducted targeted human MSOT measurements to explore the capability of MSOT to resolve lipids at the depths of the human liver in patients with previously diagnosed hepatic steatosis. Fig. 2 depicts MSOT imaging and subsequent analysis of the livers of five patients and five healthy volunteers. Fig. 2a shows an exemplary US frame recorded over the hypochondriac region of a healthy volunteer. The corresponding and coregistered MSOT image at 930 nm is provided in Fig. 2b. Likewise, Fig. 2c-d show the US and MSOT images of the same anatomic region of a patient with diagnosed hepatic steatosis. Both the SAT and liver regions are clearly delineated (dashed line/manually) in both the healthy volunteer and the patient based on the local anatomy. Two differences are apparent between the patient and the healthy volunteer: i) the lipid signal is stronger within the delineated liver region of the ‘steatosis’ patient compared to the healthy volunteer and ii) the SAT layer is thicker in the ‘steatosis’ patient.

To explore the capability of MSOT to detect lipids in human soft tissues, we analysed the high-lipid-content SAT layer. Fig. 2e-h summarize our analysis for the SAT regions of the patients and healthy volunteers. The exemplary normalized ratio spectra of both a healthy volunteer and a patient with liver steatosis feature prominent absorption-peaks at 930 nm (Fig. 2e-f). The similarity in the spectra of the SAT layers between the two groups is further apparent in the statistical analyses of the normalized ratio spectra (Fig. 2g). Furthermore, the normalized ROI/BGR ratios within the SAT region for the whole groups of healthy volunteers and hepatic steatosis patients are not significantly different (Fig. 2h; healthy volunteers 0.92 ± 0.031 vs. steatosis patients 0.9 ± 0.043, p-value = 0.6746, Cohen’s d = 0.27). Thus, as expected, MSOT is able to detect high levels of lipid signals within the SAT region in both healthy volunteers and patients with hepatic steatosis.

As a next step, we used the same principle and analysis to investigate the capability of MSOT to detect lipids within the liver region in patients with diagnosed hepatic steatosis. Fig. 2i-l illustrate the spectral analysis of the MSOT measurements of the liver regions for both healthy volunteers and patients. Comparison of exemplary normalized ratio spectra reveals higher absorption at 930 nm in the liver of the steatosis patient compared to that of the healthy volunteer, corresponding to the greater lipid content of the steatotic liver (Fig. 2i-j). Fig. 2k depicts the mean normalized ratio spectra for the whole ‘healthy’ and ‘steatosis’ human groups; the lipid-peak at 930 nm is again prominent. Moreover, the ROI/BGR ratio at 930 nm within the liver region was significantly higher in the liver steatosis group compared to the group of healthy volunteers (0.762 ± 0.146 vs. 0.219 ± 0.081, p-value = 0.011, Cohen’s d = 2.07).

**Fig. 3:**
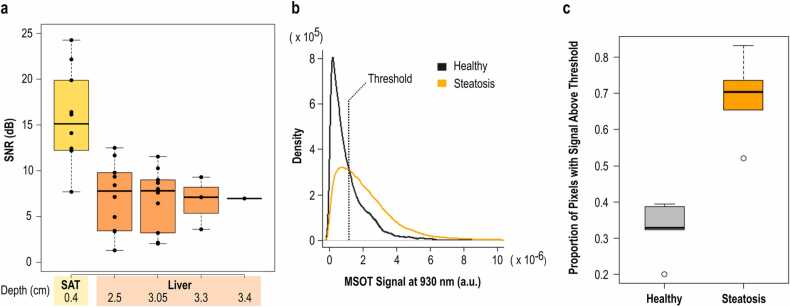
Depth analysis and pixel statistics of human data. (a) SNR in dB with increasing depth in human MSOT images at 930 nm. Each box represents the SNR for the SAT or liver segments at the noted depth for all corresponding subjects. The depths (in cm) refer to the centroid of each SAT or liver segment. (b) Density curves (histogram) for the measured optoacoustic signals at 930 nm for all pixels inside the liver ROIs of all subjects. The dashed line represents the selected threshold. (c) Proportion of pixels with optoacoustic signal above the threshold for each of the two groups (healthy volunteers vs. patients with liver steatosis, p-value < 0.001).

To confirm the capability of MSOT to resolve lipids at imaging depths of several centimeters, we analysed the effect of depth on the SNR of the signal at 930 nm from the human MSOT data (Fig. 3). Our results show a steep SNR-decrease of the 83% from the SAT (0.4 cm depth) to the first liver segment (2.5 cm depth) and a subsequent mean decrease of 24.3% for every 5 mm along the liver segments. In total, we observe an average decrease of ≈ 15% for every centimeter of depth, with a recorded SNR of 4.97 at a depth of 3.4 cm within the liver (Fig. 3a). However, the SNR at 3.4 cm is sufficient to distinguish the lipid signal from the background noise, demonstrating that MSOT is capable of detecting lipid-specific optoacoustic signals at the average depths of the human liver.

To go beyond bulk tissue analyses and gain insight into the measured OA signal within the liver at the single-pixel level, we conducted an additional statistical analysis of the pixels from the liver areas for both healthy volunteers and patients with liver steatosis. Plotting the density of OA signal intensities at 930 nm for all pixels inside the liver ROI revealed divergent distributions of OA signal intensities between the healthy volunteers and patients (Fig. 3b). In particular, the distribution within the steatosis group featured a notable tail towards higher signal intensities, suggesting the association of this subset of pixels with areas of increased lipid content. Finally, based on the presented histogram, a threshold was selected at the point of intersection between the two curves, and the proportion of pixels above this threshold was calculated for each subject. The proportion of pixels above the selected threshold was found to be significantly higher (the respective proportions test yielded a p-value < 0.001) in the patient group compared to the healthy volunteers (Fig. 3c).

**Fig. 4:**
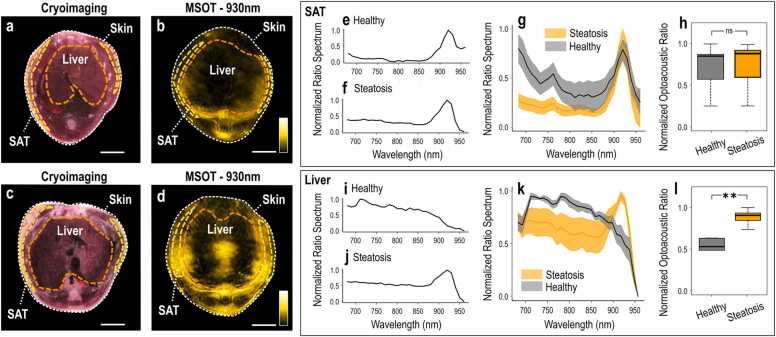
Mouse MSOT imaging and data analysis. (a) Cryoimage of a CD-fed mouse. (b) MSOT image recorded at 930 nm of the same mouse. (c) Cryoimage of an HFD-mouse. (d) 930 nm-MSOT image of the same mouse as in (c). The dashed white line marks the skin surface; the dashed yellow lines delineate the subcutaneous fat (SAT) region and the dashed orange lines the liver region. (e-f) Normalized ROI/BGR ratio spectrum within the SAT region for a CD-fed (e) and an HFD-mouse (f). (g) Mean (± standard error of the mean) normalized ROI/BGR ratio spectra for the whole cohort of the CD-fed (gray) and the HFD-fed (orange) mice. (h) Normalized ROI/BGR mean pixel intensity ratio at 930 nm within the SAT region for CD-fed (gray) and HFD-fed (orange) mice. (i-j) Normalized ROI/BGR ratio spectrum within the liver region of a CD-fed (i) and an HFD-mouse (j). (k) Mean (± standard error of the mean) normalized ROI/BGR ratio spectra for the whole cohort of the CD-fed (gray) and the HFD-fed (orange) mice. (l) Normalized ROI/BGR mean pixel intensity ratio at 930 nm within the liver region of CD-fed (gray) and HFD-fed (orange) mice. ns = non statistically significant, **p < 0.01.

To further validate the efficacy of our method for detecting hepatic steatosis in humans, we performed experiments in mice, where signal attenuation due to tissue absorption is minimal because of the shallower imaging depths. Scanned mice were either CD-fed (controls) or HFD-fed (expected to develop hepatic steatosis). Fig. 4 shows images and analyses from MSOT measurements of the mice livers and the surrounding SAT regions. Fig. 4a-d compare exemplary cryoimages and MSOT images for both a control CD-fed mouse (Fig. 4a-b) and an HFD-fed mouse with liver steatosis (Fig. 4c-d). The MSOT images shown were recorded at 930 nm, where lipids absorption is the highest. Dashed orange lines delineate the liver while dashed yellow lines delineate the SAT regions. A clear difference is visible in the size of the two mice, with the HFD-fed mouse being larger in cross-sectional diameter than the CD-fed one. This increase in size is mainly the result of an increase in the size of the SAT layer, rather than the liver. However, the optoacoustic signal intensity within the liver is visually higher in the liver of the HFD-fed mouse compared to the CD-fed control.

Spectral analysis did not indicate a significant difference in the lipid content of the SAT regions between the HFD-fed and CD-fed mice (Fig. 4e-h). Fig. 4e-f display characteristic normalized ratio spectra (680–960 nm) from the SAT regions of a ‘healthy’ mouse (Fig. 4e) and a ‘steatosis’ (Fig. 4f) mouse. The spectra are qualitatively similar, with both containing a prominent absorption peak at 930 nm, as expected for the high-lipid-content SAT region. Fig. 4g shows the mean normalized ratio spectra for the whole mouse cohort of n_4_ = 5 CD-fed healthy controls (black line) and n_3_ = 5 HFD-fed steatosis (yellow line) mice, where the peak at 930 nm is again prominent for both groups. Finally, Fig. 4h depicts the statistical analysis for the normalized ROI/BGR intensity-based ratio at 930 nm within the SAT region. The difference between the ‘liver steatosis’ and the ‘healthy’ mice groups were, as expected, not significant (0.724 ± 0.136 for HFD-fed mice vs. 0.705 ± 0.133 for CD-fed mice, p-value = 0.923, Cohen’s d = 0.06).

A similar analysis of the MSOT data from the liver region (Fig. 4i-l) reveals significant lipid accumulation in the livers of the HFD-fed mice compared to the CD-fed mice. Fig. 4i-j show exemplary normalized ratio spectra of a ‘healthy’ CD-fed and a ‘steatosis’ HFD-fed mouse. A strong peak indicative of lipid is visible at 930 nm in the ‘steatosis’ case, whereas no such peak is present in the ‘healthy’ case. Note that both spectra are characterized by higher absorption levels in the range 680–900 nm, compared to the spectra from the SAT regions (Fig. 4e-f), due to the much higher blood content of liver tissue compared to SAT. Fig. 4k displays the mean normalized ratio spectra for both groups of mice. Again, the peak at 930 nm is observed for the ‘steatosis’ group, but not for the ‘healthy’ control group. Fig. 4l illustrates the mean normalized ROI/BGR ratio at 930 nm for the liver region. In contrast to the SAT region, the difference between the two mouse groups in the liver region at 930 nm is statistically significant (0.886 ± 0.044 for HFD-fed mice vs. 0.484 ± 0.097 for CD-fed, p-value = 0.005, Cohen’s d = 2.39).

## 4 Discussion

Hepatic steatosis is a significant health problem that may lead to severe liver disease and frequently coexists with other conditions, such as obesity, type 2 diabetes, and cardiovascular disorders [34]. Non-invasive imaging can provide rich information about the pathophysiology of the disease and enable early diagnosis, but available methods come with limitations (e.g., ionizing radiation, low sensitivity, bulky equipment, varying diagnostic cut-off values) that hinder their disseminated use in research and clinical settings. Here, we demonstrated that non-invasive and label-free MSOT can detect significant differences in the spectral content of the liver in patients with steatosis and the liver of healthy volunteers, which manifest as stronger absorption around 930 nm in the liver of patients with steatosis due to the accumulation of lipids. These results suggest potential clinical and research applications of MSOT for hepatic steatosis and other disorders involving lipids.

Following previous publications on imaging lipids in the bloodstream [27] or the supraclavicular region [20], our results support the capability of MSOT to detect lipids in deeper soft tissues, such as the liver, non-invasively and without the need for exogenous contrast agents. First, by conducting targeted calculations within the SAT region of mice and humans, we showcased that MSOT indeed enables the detection of lipids based purely on the recorded spectral information from the NIR, in which lipids absorb strongly at ∼930 nm. The localization of the SAT region is validated via cryoimaging for the mouse data and via US for the human data. Second, we employ the same principle to detect lipids within the visualized liver region. Thus, MSOT provides not only localized tissue-specific lipid detection, but also detailed (spatial resolution of ≈ 100–300 µm) maps of lipid distributions within different tissues, organs, and anatomic compartments (e.g., SAT region and liver) in a single scan. The ability to resolve lipids in the liver and other soft tissues could be useful for detecting possible signs of distorted pathways of metabolism.

We demonstrated that the abovementioned unique capability of MSOT to detect lipids extends to depths necessary to analyse the human liver. We observed qualitative differences in the visual appearance and the spectral signatures of the livers of patients with steatosis compared to their healthy counterparts, with the steatotic livers being larger and featuring higher absorption at 930 nm (Fig. 2c-d, i-k). These observations in humans agreed well with the mouse data (Fig. 4c-d, i-k)), despite the much greater depth of the human liver. This qualitative observation of the spectra was confirmed by statistical analysis, which showed that absorption at 930 nm in the livers of both the preclinical and clinical steatosis groups was significantly higher than for the healthy groups (Figs. 4l and 2l; 0.886 ± 0.044 (diseased) vs. 0.484 ± 0.097 (healthy) with p-value = 0.005 in mice and 0.762 ± 0.146 (diseased) vs. 0.219 ± 0.081 (healthy) with p-value = 0.011 in humans). In other words, MSOT can provide both qualitative information about the consistency of liver tissue and semi-quantitative insights into its lipid-content. Thus, MSOT could be used not only for the detection of lipids, and thereby liver steatosis, but also for a rough estimation of the severity of the disease. Of course, more studies are needed in order to be able to provide an absolute quantification of lipids or other optoacoustically measured chromophores within the measured deep tissues.

Nevertheless, MSOT does not come without limitations. As discussed, MSOT reaches depths of approximately 3–4 cm, which is unattainable by other optical methods and sufficient for small animal imaging [35]. However, the achieved depth is poor compared to traditional techniques used for clinical liver imaging (e.g. US, CT, MRI) and cannot ensure full-depth imaging of the liver, especially in obese patients. Further development of light delivery systems or light fluence correction schemes may alleviate the effects of light attenuation and increase imaging and spectral unmixing quality with increasing depths. Moreover, spectral unmixing is currently done on a per-pixel basis and the quality is highly dependent on intrinsic (e.g., breathing, arterial pulsation) or extrinsic (e.g. hand movements during scanning) motion. For these reasons, novel motion correction schemes and spectral unmixing algorithms have been developed in order to enable the precise visualizations of chromophores in deep tissues [36], [37], [38]. Finally, some steps of the MSOT data analysis process (e.g., segmentation, interpretation) might be time-consuming or labor-intensive, especially in the case of large (e.g., hundreds or thousands of images) or noisy datasets. Therefore, several automatic segmentation or image improvement techniques have been developed, even if herein, due to the small number of participants and the high data quality, we opted for a manual segmentation approach [39], [40].

However, the grading of hepatic steatosis is not routine; it is mainly performed using histology, i.e., using the NAFLD Activity Score (NAS), or sometimes by means of MRI or FibroScan©. In our patient cohort, no liver biopsies were performed and, thus, no histological assessments are available. Future studies are needed in order to explore the capability of MSOT to assess hepatic steatosis at different stages, as well as investigate possible correlations of MSOT with other imaging modalities or histology.

While the current preliminary translational study is the first to successfully apply hand-held MSOT imaging for the detection of hepatic steatosis in humans, it includes only a limited number of subjects. Furthermore, even if hand-held MSOT technology reaches unprecedented depths of several centimeters compared to other light-based imaging approaches (e.g., fluorescence imaging), it cannot provide full-depth imaging of the human liver (≈ 8–10 cm), especially in obese patients. Future targeted and more extended studies will seek to i) assess the specificity, sensitivity and accuracy of MSOT technology for the assessment of hepatic steatosis, ii) investigate the possible differences among different types of fatty liver disease, and iii) further advance MSOT towards clinical translation for liver diagnostics. Our recordings were further supported by corresponding mouse data and MSOT provided the expected results in the scanned anatomic compartments, as validated by cryoimaging and medical US imaging. Thus, the proposed MSOT-based method generates new possibilities for investigating the pathophysiology of hepatic steatosis and demonstrates translational potential with possible implications for future clinical diagnostics of the disease in selected patients.

## Funding

This project has received funding from the 10.13039/501100000781European Research Council (ERC) under the European Union’s Horizon 2020 research and innovation programme under grant agreement No 694968 (PREMSOT) and was supported by the 10.13039/100010447DZHK (German Centre for Cardiovascular Research; FKZ 81Z0600104), the 10.13039/501100013295Helmholtz Zentrum München funding program “Physician Scientists for Groundbreaking Projects” and by a Clinical Leave Stipend from the German Center of Infection Research (DZIF, grant TI07.001).

## CRediT authorship contribution statement

**Nikolina-Alexia Fasoula:** Conceptualization, Methodology, Formal analysis, Investigation, Writing – original draft. **Angelos Karlas:** Conceptualization, Methodology, Formal analysis, Investigation, Writing – original draft, Visualization, Funding acquisition. **Olga Prokopchuk:** Methodology, Investigation, Writing – original draft, Funding acquisition. **Nikoletta Katsouli:** Methodology, Formal analysis, Visualization. **Michail Bariotakis:** Methodology, Formal analysis, Writing – original draft, Visualization. **Evangelos Liapis:** Methodology, Writing – original draft. **Anna Goetz:** Methodology. **Michael Kallmayer:** Methodology, Writing – review & editing. **Josefine Reber:** Methodology, Investigation, Writing – original draft. **Alexander Novotny:** Writing – original draft. **Helmut Friess:** Supervision. **Marc Ringelhan:** Writing – original draft. **Roland Schmid:** Supervision. **Hans-Henning Eckstein:** Supervision. **Susanna Hofmann:** Writing – review & editing, Supervision. **Vasilis Ntziachristos:** Conceptualization, Writing – review & editing, Supervision, Funding acquisition.

## Declaration of Competing Interest

V.N. is a founder and equity owner of sThesis GmbH, iThera Medical GmbH, Spear UG, and i3 Inc. All other authors declare that they have no competing interests.

## Data Availability

Data will be made available on request.
